# Efficacy and cost-effectiveness of a blended cognitive behavioral therapy for depression in Spanish primary health care: study protocol for a randomised non-inferiority trial

**DOI:** 10.1186/s12888-018-1638-6

**Published:** 2018-03-23

**Authors:** Mª Dolores Vara, Rocío Herrero, Ernestina Etchemendy, Macarena Espinoza, Rosa Mª Baños, Azucena García-Palacios, Guillem Lera, Blanca Folch, Vicente Palop-Larrea, Pilar Vázquez, Manuel Franco-Martín, Annet Kleiboer, Heleen Riper, Cristina Botella

**Affiliations:** 1 Department of Personality, Evaluation and Psychological Treatment, University of Valencia, Valencia, Spain; 2 Department of Basic Psychology, Clinical and Psychobiology, Jaume I University, Castellón, Spain; 3 Department of Personality, Evaluation and Psychological Treatment, University of Zaragoza, Zaragoza, Spain; 4 CIBER Fisiopatología Obesidad y Nutrición (CIBEROBN), Instituto Carlos III, Madrid, Spain; 5 Hospital Universitario de la Ribera. Alzira (Valencia), Valencia, Spain; 6 Complejo Asistencial de Zamora, Zamora, Spain; 7 Section Clinical Psychology, Vrije Universiteit Amsterdam and EMGO+ Institute for Health Care and Research, Amsterdam, the Netherlands; 8 Department of Psychiatry, VU University Medical Centre and EMGO+ Institute for Health Care and Research, Amsterdam, the Netherlands

**Keywords:** Blended treatment, Cognitive behavioral therapy, Depression, Internet-based treatment, Primary health care, Randomised non-inferiority trial

## Abstract

**Background:**

Data from primary health care in Spain show a high prevalence of the major depressive disorder. Blended treatment (combination of face-to-face and online components) seems to be a very promising tool for the optimization and dissemination of psychological treatments in a cost-effective form. Although there is growing data that confirm the advantages of blended therapies, few studies have analyzed their application in regular clinical practice. The objective of the present paper is to describe the protocol for a clinical study aimed at exploring the clinical and cost-effectiveness of a blended cognitive behavioral therapy (b-CBT) for depression, compared to treatment as usual (TAU) in a primary health care setting.

**Methods:**

A two-arm randomised controlled non-inferiority trial will be carried out, with repeated measures (baseline, 3 months, 6 months, and 12 months) under two conditions: b-CBT and TAU. The b-CBT program will consist in three face-to-face sessions and eight online sessions. The TAU is defined as the routine care delivered by the general practitioner for the treatment of depression in primary care. The primary outcome is a symptomatic change of depressive symptoms on the patient-health questionnaire (PHQ-9). Other secondary outcomes will be considered (e.g., quality of life, treatment preference). All participants must be 18 years of age or older and meet the diagnostic criteria for major depressive disorder according to the Diagnostic and Statistical Manual of Mental disorders 4th edition. 156 participants will be recruited (78 per arm).

**Discussion:**

It is expected that b-CBT is clinically non-inferior when compared to TAU. This is the first study in Spain to use a b-CBT format in primary and specialized care, and this format could be an efficacious and cost-effective therapeutic strategy for the treatment of depression.

**Trial registration:**

ClinicalTrials.gov NCT02361684. Registered on 8 January 2015. Currently recruiting participants.

## Background

Depression is a highly prevalent disorder with considerable personal and social costs in terms of quality of life, wellbeing, and economic effects [1–4]. In this regard, the World Health Organization (WHO) predicts that depression will be one of the three leading causes of the burden of disease by 2030 [5, 6]. Even though there are effective treatments for this emotional disorder (pharmacotherapy, psychotherapy or both) [7–9], these treatments fail to reach the growing number of people who need them [10]. Data from primary health care in Spain reveal a high prevalence of mental disorders (with depression at the top) [11–13] and an important gap between the number of people suffering from depression and the rate of treatment received [14, 15]. Therefore, it becomes necessary to incorporate new approaches into the traditional ways of providing psychological treatments, in order to effectively respond to this need [10, 16]. Internet-based treatments are a cost-effective alternative that can improve treatment dissemination, becoming a useful resource to address the gap between treatment demand and supply. In recent years, several studies have assessed the effectiveness and acceptability of computer-based treatments for depression, obtaining positive results [17–19]. Regarding efficacy, several systematic reviews have found that online treatments are more effective when guided (ranging from automated reminders to encouragement and feedback by email, text messages, or brief telephone calls) [20, 21], highlighting the positive association between therapist contact and improvement rates [22]. Some data also show that guided self-help and face-to-face treatments do not significantly differ in their effectiveness [23–25].

Due to the benefits of including therapist support in Internet-delivered treatments, and seeking the best approach in terms of cost-efficacy, the “blended treatment” format emerges as a good alternative to address the growing need for access to evidence-based psychological treatments for mental disorders. Blended treatment usually refers to the combination of face-to-face and online components [26]. This format combines the best qualities of each therapeutic approach: broader dissemination, less therapist time, lower health services costs, and direct therapist-patient support and guidance, which may lead to greater adherence. There are previous experiences with this treatment format, with positive outcomes in terms of efficacy, adherence, and relapse prevention [27–31].

Despite the above, very little research has focused on blended treatments in regular clinical practice (e.g., [32]). If the goal is to diminish the burden of mental disorders and decrease the gap between treatment demand and true access to psychological support, it is necessary to assess the efficacy, feasibility, and cost-effectiveness of these types of treatments. Therefore, the aim of the present paper is to describe the protocol for a randomised controlled non-inferiority trial that compares the clinical and cost-effectiveness of blended cognitive behavioral therapy (b-CBT) for adults suffering from major depressive disorder (MDD) and treatment as usual (TAU) in a primary health care setting.

## Methods

### Aim

The aim of the study is to explore the clinical and cost-effectiveness of a b-CBT for depression, compared to TAU, in primary care in Spain. This trial is part of the E-COMPARED project [European 7FP, N° Agreement; 603,098], which includes several randomised controlled non-inferiority trials in both primary care and specialized care in eight European countries, in an effort to obtain clinically significant data.

The hypothesis is that b-CBT will be at least as effective as the TAU condition, defined as the routine care that patients receive in primary care in Spain when diagnosed with depression.

### Study design

A two-arm randomised controlled non-inferiority and cost-effectiveness trial will be carried out, with repeated measures (baseline, 3 months, 6 months, and 12 months) and two conditions: b-CBT and TAU. The study will be conducted following the CONSORT statement (Consolidated Standards of Reporting Trials, http://www.consort-statement.org) [33, 34], the CONSORT-EHEALTH guidelines [35], and the Recommendations for Interventional Trials (SPIRIT) [36, 37].

### Sample size

The sample size calculation is based on the non-inferiority design and calculated for the primary clinical outcome: depression symptoms (3 months after baseline). Following Cohen [38], f = 0.20 represents an effect size of small magnitude, which is a conservative estimate of the subjective minimal important difference noted by patients. Taking into account the E-COMPARED project as a whole, and considering an alpha of 0.05 and a statistical power of 0.90, the total sample size required to warrant these conditions is 1052 participants. Based on the literature on Internet-based treatments, a 30% dropout rate is expected [39]. Thus, the total number of participants to be recruited will be 1200, 150 patients in each country involved in the project. However, in the case of the trial in Spain, we intend to include 156 participants (78 per arm). This sample size was calculated assuming that there is no difference between standard and blended depression treatment, considering a statistical power of 0.80, and that the lower limit of a one-sided 95% confidence interval will be above a non-inferiority limit of − 0.4. A margin of 0.4 was judged acceptable, as this range of a small to moderate difference in effect size will not result in clinically important differences.

### Study population, recruitment, and eligibility criteria

Participants who report depression to their general practitioner (GP) will be consecutively invited to participate in the trial. This recruitment will be implemented in primary care centers belonging to the Hospital of the Rivera in Valencia, and the Provincial Hospital of Zamora. All interested participants must give written informed consent to take part in the trial. To confirm inclusion/exclusion criteria (see Table 1), a telephone screening interview will be carried out by a psychologist from the University of Valencia and the University of Castellón, using the MINI International Neuropsychiatric Interview for diagnosis version 5 (M.I.N.I. 5.0) [40–42]. If the participant meets the eligibility criteria, after completing the baseline measurement, randomization will be conducted.

**Table 1:** Inclusion/exclusion criteria

Inclusion Criteria	Exclusion criteria
Minimum age of 18 years old	Presence of serious psychiatric comorbidities (substance dependence, bipolar affective disorder, obsessive compulsive disorder, psychotic illness)
Meeting the DSM-IV diagnostic criteria for Major Depressive Disorder	High risk for suicide
Ability to understand and read Spanish	Receiving psychological treatment for depression at the time of recruitment
Access to Internet and having an email address	An increase and/or change in the pharmacological treatment (in the case of receiving it) during the study period
Providing written informed consent	Medical disease that prevents the participant from carrying out the psychological treatment

**Fig. 1:**
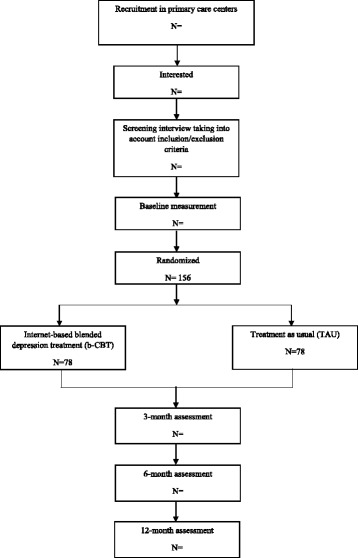
Patient flow diagram

An independent researcher from VU University in Amsterdam will create an allocation scheme using a block randomization design (variable block sizes) with a computerized random number generator (Random Allocation Software) at an allocation ratio of 1:1. Participants will be assigned to two conditions: b-CBT or TAU. The condition to which they are assigned will be communicated to the participants by telephone. Blinding for the treatment is not possible because it will be clear to both therapists and patients whether the treatment is blended or not. The follow-up measurements will be administered online and by telephone (3 months, 6 months, and 12 months). Figure 1 shows the study flow chart.

### Ethics

The study follows the guidelines of the Helsinki Declaration [43]. All the researchers will follow the guidelines for Good Clinical Practice [44]. As noted, all the participants will be volunteers, and they will not receive any compensation for their participation. They will sign the informed consent once the study and its conditions have been explained. Participants will be able to withdraw from the study at any time, without giving any reason and with no consequences. The study is registered in the United States National Institute of Health Registration System (http://www.clinicaltrials.gov) with Clinical Trials Registration Number: NCT02361684, https://clinicaltrials.gov/ct2/show/NCT02361684.

### Interventions

#### Blended cognitive behavioral therapy (b-CBT)

Cognitive Behavioral Therapy (CBT) is the most commonly used and recommended treatment due to its favorable clinical outcomes in depression [45, 46]**.** It usually focuses on four psychological components: 1) psycho-education, 2) behavioral activation, 3) cognitive therapy, and 4) relapse prevention. All E-COMPARED interventions must provide these four core components, and they can additionally provide two extra components.

All E-COMPARED project interventions combine individual CBT delivered through face-to-face sessions (FTF) and online sessions. The ratio used is one FTF session per three sessions delivered over the Internet. The b-CBT will be provided by therapists (clinical psychologists**)** who will receive special training in blended CBT and how to deliver it. Therapists will receive a manual with the corresponding procedure and content for each FTF session. All therapists will have a minimum of two years of work experience in Spanish mental health care.

Regarding the intervention protocol, this b-CBT program will be composed of three 45-min face-to-face sessions and eight online sessions in 10 weeks.

#### Face-to-face sessions

The first FTF session will be held at the beginning of the treatment. It will focus on clarifying the instructions for the use of the online platform, and motivation for life changes will be addressed. In the middle of the intervention (module 6), participants will receive the second FTF session. The purpose of this session is to resolve doubts about previous therapeutic contents and reinforce commitment and adherence to treatment. The last FTF session is held at the end of the entire program. This final session is dedicated to analyzing possible difficulties and presenting and discussing relapse prevention with the participants. Between the FTF sessions, the following four online sessions will be implemented.

#### Online treatment platform

The online part of this intervention corresponds to the “Smiling is Fun” program [47, 48]. It is an Internet-delivered, multimedia, interactive, self-help and self-applied program for emotional disorders (e.g., depression). It follows a transdiagnostic perspective and is based on CBT techniques. It is mainly designed to learn and practice adaptive ways to cope with depression and daily problems. It was developed within Project OPTIMI (Online Predictive Tools for Intervention in Mental Illness), funded by the VII Program Framework of the European Union. The program includes six treatment components (motivation, psycho-education, behavioral activation strategies, cognitive therapy, positive psychology strategies, and relapse prevention) addressed through eight modules. Each module and its specific objectives are shown in Table 2.

**Table 2:** Modules and objectives of “Smiling is Fun”

Module	Objective
(1) Motivation for change	Analyze the advantages and disadvantages of changing, emphasizing the importance of being motivated.
(2) Understanding emotional problems	Recognize and understand emotional problems.
(3) Learning to move on	Teach the importance of “moving on” to acquire a proper level of activity and involvement in life.
(4) Learning to be flexible	Teach a more flexible way of thinking, interpreting situations, and learning to think about different alternatives.
(5) Learning to enjoy	Generate positive emotions, promoting the involvement in pleasant and significant activities and contact with other people.
(6) Learning to live	Understand the importance of identifying the individual’s own psychological strengths and selecting and carrying out meaningful activities linked to values and goals in life.
(7) Living and learning	Develop an action plan to boost individual psychological strengths.
(8) From now on, what else…?	Go on and strengthen what has been learned during the program.

The program also includes two interactive tools that accompany the patients during the intervention: 1) The *Activity diary* provides feedback about mood, activities performed, and their relationship, and it also shows the mood benefits of being involved in meaningful activities; 2) The *“How am I?” section* offers a set of graphs and feedback to chart the user’s progress in terms of level of activity, emotional discomfort, and intensity of positive and negative emotionality.

Moreover, the Internet platform includes a mobile phone component that enables daily ecological momentary assessment (EMA) of the participant’s mood state (e.g., *What is your mood right now?*), cognitions (e.g., *How much are you worrying at the moment?*), activities (e.g., *How much did you enjoy the activities today?*), social interaction (e.g., *How much were you involved in social interactions today?*), and sleep (e.g., *How well did you sleep last night?*). All the EMA measures will be time and day stamped.

#### Treatment as usual (TAU)

TAU is defined as the routine care delivered by the general practitioner for the treatment of depression in primary care. The type of treatment can vary depending on the GP’s opinion and the severity of each patient. In Spain, the intervention generally consists of antidepressant medication. The specific type of treatment implemented will be monitored.

### Adherence

If participants do not enter the online platform (for more than 15 days), they will receive emails reminding them of the importance of reviewing the modules and encouraging them to do the homework tasks. A professional platform will be used to send these emails (www.psicologiaytecnologia.com). In addition, the therapists will call the participants to schedule the FTF sessions.

### Measures

The study measures and assessment times (online and by phone) are summarized in Table 3.

**Table 3:** The study measures and assessment times

Variable	Instrument	Screening baseline		3 months		6 months		12 months	
		Online	Phone	Online	Phone	Online	Phone	Online	Phone
Questions for patients									
Demographics and history of mental health treatments			x						
Diagnostic interview	M.I.N.I.		x						x
Symptoms of depression	PHQ-9 QUIDS SR-16	x		x		x		x	
Quality of life	EQ-5D-5 L AQol	x		x		x		x	
Health care uptake and productivity at work	TiC-P		x		x		x		x
Treatment preference			x						
Patient expectancy of treatment	CEQ	x							
Working alliance	WAI-SF			x					
Technology alliance	TAI-OT^a^			x					
Client satisfaction	CSQ			x					
Satisfaction with the online program	SUS^a^			x					
Questions for therapists									
Working alliance	WAI-SF			x					
Satisfaction with the online program	SUS^a^			x					

*M.I.N.I* MINI International Neuropsychiatric Interview, *PHQ-9* Patient Health Questionnaire-9, *QUIDS SR-16* Quick Inventory of Depressive Symptomatology-16, *EQ-5D-5 L* EuroQol 5D, *AQol* Assessment of Quality of Life, *TiC-P* Trimbos and iMTA Questionnaires on Costs Associated with Psychiatric Illness, *CEQ* Credibility and Expectancy Questionnaire, *WAI-SF*, Working Alliance Inventory, *TAI-OT* WAI Online Therapy, *CSQ* Client Satisfaction Questionnaire, *SUS* System Usability Scale

^a^This instrument will be administered in the b-CBT condition

### Primary outcome measures

#### Symptoms of depression

Patient Health Questionnaire-9 (PHQ-9) [49] will be used as a primary outcome measure. It is a 9-item questionnaire that can be used to screen and diagnose patients with depressive disorders. The nine items are each scored on a 0–3 scale. Total scores range from 0 to 27; higher scores indicate more severe depression. The PHQ-9 has been shown to have good psychometric properties [50].

### Secondary outcome measures

#### Diagnostic interview

The MINI International Neuropsychiatric Interview version 5.0 (M.I.N.I. 5.0) [40–42] will be used at screening to assess current depression and current comorbid disorders. This measure is a structured diagnostic interview based on the Diagnostic and Statistical Manual of Mental Disorders 4th edition (DSM-IV) and on International Classification of Diseases-10 (ICD-10) criteria.

#### Severity of depression

The Quick Inventory of Depressive Symptomatology-16 (QIDS SR-16) [51, 52]. This scale is a 16-item self-report that assesses the severity of depression and nine symptom domain criteria (sleep, sad mood, appetite-weight, concentration-decision making, self-view, thoughts of death or suicide, general interest, energy level, and restlessness/agitation) that define a major depressive episode according to the DSM-IV.

#### Quality of life

It will be measured with the EuroQol 5D (EQ-5D-5 L) [53–55] and the Assessment of Quality of Life (AQol) questionnaire [56]. The former is a self-report questionnaire that measures health-related quality of life and enables conversion to utility scores to calculate Quality-Adjusted Life-Years (QALYs) [57, 58]. This scale is composed of five dimensions with 5 items related to anxiety or depression: mobility, self-care, ordinary activities, discomfort, and mood state. The AQol consists of 20 items covering five dimensions that measure the “utility” of health states: illness, independent living, social relationships, physical senses, and psychological well-being. In both instruments, the five categories are measured in a range from “no problems” to “a lot of problems”.

### Cost measures

#### Health care uptake and productivity at work

This will be evaluated with the Trimbos and iMTA Questionnaire on Costs Associated with Psychiatric Illness (TiC-P) [59]. It is an 11-item self-report questionnaire with two different parts that can be administered separately. Part I assesses the participant’s health care and medication use. Part II measures lost productivity costs resulting from absenteeism (being absent from work because of illness) and presenteeism (being present at work while ill, which may lead to reduced efficiency).

### Other measures

#### Demographic variables and history of mental health treatments

Personal data that include information such as age, gender, country of birth, education level, and treatments received for psychological problems.

#### Preference for treatment

The participants will indicate their treatment preference, choosing between the b-CBT and TAU options (“If you had the chance to choose your depression treatment, which one would you prefer to receive?”).

#### Patient expectancy of treatment

This variable will be measured with the Credibility and Expectancy Questionnaire by Devilly and Borkovec (CEQ) [60]. This 6-item self-report instrument measures treatment credibility and patient expectations of improvement.

#### Therapeutic alliance

This will be assessed using the short version of the Working Alliance Inventory (WAI-SF) [61]. This inventory is a 12-item (for patient) and 10-item (for therapist) self-report questionnaire with responses given on a 5-point Likert scale ranging from 1 (never) to 5 (always), considering three dimensions: (1) therapeutic goals, (2) tasks, and (3) bond. The alliance between the patient and technologies will be assessed with the WAI Online Therapy questionnaire (TAI-OT) developed by Labpsitec (http://www.labpsitec.uji.es/esp/index.php).

#### Client satisfaction

The Client Satisfaction Questionnaire (CSQ) [62, 63] will be used to assess overall patient satisfaction with health and human services. It consists of 8 items measured on a 4-point scale with total scores ranging from 8 to 32 points.

#### Satisfaction with the online program

To assess the overall usability of the online program, the System Usability Scale (SUS) [64] will be used. It is a 10-item scale, measured on a 5-point scale ranging from strongly disagree to strongly agree. Total SUS scores range from 0 to100. The questionnaire was found to be reliable and robust [65].

### Ecological momentary assessment (EMA)

#### Daily functioning

During the first and last week of treatment, twice a day (morning and evening), participants will receive a set of questions on their mobile phones about their mood state (e.g., What is your mood right now?), cognitions (e.g., How much are you worrying at the moment?), activities (e.g., How much did you enjoy activities today?), social interaction (e.g., How much were you involved in social interactions today?), and sleep (e.g., How well did you sleep last night?). They will also receive them once a week during treatment on a random day. In all cases, participants will rate their answers on a 0–10 Likert scale.

### Analysis

Multiple imputation will be used to deal with missing data. The primary statistical analyses will be group comparisons of improvements in depressive symptoms. An analysis of covariance (ANCOVA) model with completers’ data will be used for this purpose. Intention-to-treat analyses will be used in sensitivity analyses to increase confidence in the results obtained. It is hypothesized that blended depression treatment will be equally as effective as care-as-usual. A non-inferiority margin and the smallest clinically acceptable difference will be considered to exist when the two-sided 95% confidence interval (the range of plausible differences between the two treatments) lies entirely above the standard mean difference of 0.20.

## Discussion

The purpose of the present study protocol is to explore the clinical effectiveness of a b-CBT for depression, compared to treatment as usual, in Spanish primary care settings. It is expected that the combination of FTF with online sessions will be at least as effective as usual care in improving depressive symptoms. Moreover, it is expected that the blended format, although somewhat more demanding in terms of commitment of clinician hours than a completely self-applied treatment, will be an adequate way to deliver empirically validated treatments, combining two key needs: the need for access and treatment dissemination and the need for therapist support during treatments. To our knowledge, this is the first study in Spain to use a b-CBT format in primary care settings, and this format could be an efficacious and cost-effective therapeutic option for the treatment of depression.

One of the strengths of the present study is the inclusion of ecological momentary assessments throughout the treatment period. This can be a relevant source of information about participants’ evolution in terms of improvement and worsening and their association with specific therapeutic components. Even though these data may not be sufficient to analyze the isolated impact of each program module (given the study design implemented here), they will be a significant reflection of the real usefulness and daily impact of this intervention on the quality of life of the participants involved.

Another strength of the study is its focus on primary health care, given the need to assess the real efficacy, feasibility, and cost-effectiveness of blended treatments in these settings, with the hope that they can become a viable resource to respond to the increasing demand for psychological care.

Regarding possible limitations, we can expect some barriers and/or negative attitudes from the GPs about recommending this treatment to their patients, and high dropout rates in the treatment group can be expected (around 30%), based on the literature [e.g., 39].

If the results of this study show the expected efficiency and efficacy of b-CBT, this could be a first step in the implementation of these treatment models in primary care as a potential solution to different problems the Healthcare system is facing at this time (such as the need to reduce costs and increasing demand from patients). In conclusion, positive results of this study could have a significant impact on primary care settings in the National Health System in Spain and in society in general.

### Trial status

The study commenced recruitment in February 2015 and is currently recruiting.

## Abbreviations

ANCOVA: Analysis of covariance
AQol: Assessment of Quality of Life
b-CBT: Blended cognitive behavioral therapy
CBT: Cognitive Behavioral Therapy
CEQ: Credibility and Expectancy Questionnaire
CONSORT: Consolidated Standards of Reporting Trials
CSQ: Client Satisfaction Questionnaire
DSM-IV: Diagnostic and Statistical Manual of Mental Disorders 4 th edition
EMA: ecological momentary assessment
EQ-5D-5 L: EuroQol 5D
FTF: Face-to-face sessions
GP: General practitioner
ICD-10: International Classification of Diseases-10
M.I.N.I. 5.0: MINI International Neuropsychiatric Interview version 5.0
MDD: Major depressive disorder
PHQ-9: Patient Health Questionnaire-9
QALYs: Quality-Adjusted Life-Years
QIDS SR-16: Quick Inventory of Depressive Symptomatology-16
SPIRIT: Recommendations for Interventional Trials
SUS: System Usability Scale
TAI-OT: WAI Online Therapy
TAU: treatment as usual
TiC-P: Trimbos and iMTA Questionnaires on Costs Associated with Psychiatric Illness
WAI-SF: Working Alliance Inventory
WHO: World Health Organization
